# Prevalence and Risk Factors of SARS-CoV-2 Infection among Parturients and Newborns from Luanda, Angola

**DOI:** 10.3390/pathogens10111494

**Published:** 2021-11-16

**Authors:** Cruz S. Sebastião, Paolo Parimbelli, Manuela Mendes, Euclides Sacomboio, Joana Morais, Jocelyne Neto de Vasconcelos, Miguel Brito

**Affiliations:** 1 Centro de Investigação em Saúde de Angola (CISA), Caxito, Angola; cruzdossantos10@gmail.com (C.S.S.); jocelyne.vasconcelos@gmail.com (J.N.d.V.); 2 Instituto Nacional de Investigação em Saúde (INIS), Luanda, Angola; euclissacomboio@hotmail.com (E.S.); jfm.morais9@gmail.com (J.M.); 3 Instituto Superior de Ciências da Saúde (ISCISA), Universidade Agostinho Neto (UAN), Luanda, Angola; 4 Maternidade Lucrécia Paim, Ministério da Saúde, Luanda, Angola; kinzenze@libero.it (P.P.); manuelamendes53@gmail.com (M.M.); 5 Faculdade de Medicina, Universidade Agostinho Neto, Luanda, Angola; 6 Health and Technology Research Center, Escola Superior de Tecnologia da Saúde de Lisboa, Instituto Politécnico de Lisboa, 1990-096 Lisboa, Portugal

**Keywords:** SARS-CoV-2, COVID-19, pregnant women, maternal breastfeeding, newborn screening, Luanda, Angola

## Abstract

SARS-CoV-2 emerged in China in December 2019, creating a massive public health concern. Although previous studies have identified SARS-CoV-2 in pregnant women, the possibility of transmission to newborns remains uncertain. Herein, we investigated SARS-CoV-2 infection and risk factors among parturients and newborns. This was a cross-sectional study carried out with 3633 parturients from Luanda, Angola, between January and April 2021, with an age ranging from 13 to 48 years. SARS-CoV-2 infection of the parturients was further confirmed with RT-PCR after COVID-19 Ag Rapid Testing. About 0.4% of parturients tested positive on the day of delivery. Surprisingly, parturients from urbanized areas (OR: 0.18, *p* = 0.025) had a low chance of infection. None of the newborns tested positive in the first 24 h after birth, while one (9.1%, 1/10) of the newborns tested positive with pharyngeal swabs seven days after birth. However, whether the case was due to vertical transmission from mother to child remains to be confirmed. The mother’s residence, education level, antenatal follow-up, and delivery category were related to SARS-CoV-2 transmission (*p* < 0.05). Our findings showed a relatively low SARS-CoV-2 infection from parturients to newborns, regardless of the severity of the maternal disease. Furthermore, these findings are an early assessment of COVID-19 cases in late pregnancy, which could indicate the need for intensive management of SARS-CoV-2 infection among parturients in Angola. Further studies are needed on the consequences of SARS-CoV-2 among pregnant women and neonates from Angola.

## 1. Introduction

The new coronavirus, known scientifically as severe acute respiratory syndrome of coronavirus 2 (SARS-CoV-2), was first identified in Wuhan, one of China’s provinces, in December 2019 [1,2]. Since then, SARS-CoV-2 has spread rapidly and is now considered to be one of the biggest public health concerns. By the end of May 2021, about 166 million cases and more than 3 million deaths were related to SARS-CoV-2 infection worldwide [3]. During the same period, the Ministry of Health of Angola reported more than 32,000 cases and 700 deaths related to SARS-CoV-2 infection in Angola [3].

Viral pneumonia is known worldwide as an important concern among pregnant women, especially in low- and middle-income countries (LMICs) [4,5,6]. Furthermore, previous studies have shown that maternal pneumonia can cause unfavorable obstetric outcomes, including restriction of intrauterine growth, rupture of membranes, premature birth, intrauterine fetal death, and neonatal death [7,8,9]. In addition, pregnant women may have a worrying clinical picture when infected with other coronaviruses such as SARS-CoV or MERS-CoV [10,11].

Little information is currently available on the transmission of SARS-CoV-2 in newborns [12,13]. Therefore, in this study, we investigated the prevalence of SARS-CoV-2 in parturients and the risk factors that may be related to SARS-CoV-2 transmission to newborns in Luanda, the capital city of Angola. The results of this study can support the Ministry of Health of Angola in defining strategies to be provided to pregnant women infected with SARS-CoV-2 in Angola.

## 2. Materials and Methods

### 2.1. Study Design and Setting

A cross-sectional study was carried out with 3633 parturients from Lucrécia Paim maternity, located in Luanda, the capital city of Angola, between January and April 2021. Lucrécia Paim maternity is a public health unit and reference center for providing health care to pregnant women and newborns from all provinces of Angola. The study was reviewed and approved by the national ethics committee of the Ministry of Health of Angola (approval no. 35/2020) and by the general director of Lucrécia Paim maternity (approval no. 840/GDG/MLP/2020). In addition, informed consent was presented to participants where they, their parent or legal guardian, freely accepted to participate in the study and follow the newborn. All data used in the analyses were coded and restricted access was given only to the research team in order to anonymize the personal data of the studied participants.

### 2.2. Sample Collection and Testing

A structured questionnaire prepared by the research team was used to collect sociodemographic, epidemiological, and clinical data before proceeding with the test to detect SARS-CoV-2 infection. After, a nasopharyngeal smear sample was collected from all participants before labor began. The collected samples were used for the screening of the SARS-CoV-2 antigen using a COVID-19 Ag Rapid Test Device kit (Panbio, Seoul, Korea, Abbott) [14]. The entire technical procedure and interpretation of the results were carried out according to the instructions provided by the manufacturer [14]. This test allows for rapid in vitro diagnostics for the qualitative detection of the SARS-CoV-2 antigen. The research team worked with the health unit’s clinical staff to guarantee clinical assistance in case of any positive results for SARS-CoV-2. The parturients who tested negative had normal care according to the maternity’s protocol. On the other hand, parturients who tested positive for SARS-CoV-2 were isolated in a section established for the management of pregnant women infected with SARS-CoV-2 in the Lucrecia Paim maternity unit. Newborns of mothers positive for SARS-CoV-2 were also screened for SARS-CoV-2 infection in the first 24 h of life, repeating the test after seven days. In addition, a new sample was collected from all reactive participants and sent to the SARS-CoV-2 testing reference center of the Angolan Ministry of Health to perform real-time reverse transcriptase-polymerase chain reaction (RT-PCR) for SARS-CoV-2 nucleic acid of nasopharyngeal swabs in order to confirm SARS-CoV-2 infection.

### 2.3. Statistical Analysis

Statistical analysis was performed using SPSS version 25 (IBM SPSS Statistics, Armonk, NY, USA). Frequencies and percentages were part of the descriptive analysis. The normally distributed data are presented as mean and standard deviation (SD). The variables were categorized, and the chi-square (X^2^) and logistic regression tests were applied to verify the relationship between the categorical variables. In addition, the odds ratio (OR) and 95% confidence intervals (CIs) were calculated to determine the strength and direction of relationships. All reported *p*-values are two-tailed and were deemed statistically significant when *p* < 0.05.

## 3. Results

### 3.1. Prevalence and Factors Related to SARS-CoV-2 Infection

The putative characteristics related to SARS-CoV-2 among parturients from Luanda are summarized in Table 1. This study included a total of 3633 parturients screened for SARS-CoV-2 between January and April 2021. Age ranged from 13 to 48 years, with the mean age being 28 ± 7 years old. Parturients that were residents of urbanized areas (50.3%, 1826/3633), with a high educational level (68.2%, 2476/3633), who were unemployed (71.5%, 2599/3633), and who had antenatal care follow-up (88.5%, 3214/3633) were the most prevalent in the studied population. A total of 13/3633 (0.4%) of the parturients tested positive for SARS-CoV-2 using the COVID-19 Ag Rapid Test. Additionally, all the positive SARS-CoV-2 parturients also tested positive in the RT-PCR assay. A significant relationship was observed between residence area and SARS-CoV-2 prevalence (*p* = 0.012). Moreover, no relationship was observed between age, educational level, occupation, or antenatal care follow-up with the SARS-CoV-2 positivity (*p* > 0.05). Although not significant, the risk of SARS-CoV-2 infection was higher in parturients aged between 20 and 40 years (OR: 1.44 (95% CI: 0.19–11.1), *p* = 0.727) and parturients with a high educational level (OR: 1.56 (95% CI: 0.43–5.68), *p* = 0.500). On the other hand, parturients from urbanized areas (OR: 0.18 (95% CI: 0.40–0.81), *p* = 0.025), who were employed (OR: 0.46 (95% CI: 0.10–2.06), *p* = 0.307), and who had antenatal follow-up (OR: 0.72 (95% CI: 0.16–3.24), *p* = 0.665) had a low likelihood of SARS-CoV-2 infection compared to the others.

### 3.2. Characteristics of the Mother with SARS-CoV-2 and Neonates Born to SARS-CoV-2-Infected Mothers

The characteristics of the mother with SARS-CoV-2 infection and neonates born to SARS-CoV-2-infected mothers are provided in Table 2. The mean age of parturients infected with SARS-CoV-2 was 30 ± 5 years. About 38.5% of parturients had morbidity during pregnancy. Eclampsia (7.7%), urinary tract infections (7.7%), and malaria (15.4%) were the most frequent morbidities observed in this studied population. Vaginal delivery was observed in 69.2% and cesarean delivery was observed in 30.8% of the population. Among SARS-CoV-2-positive parturients, 69.2% were symptomatic at the time of delivery, and subsequently asymptomatic at day 7 after delivery. The most frequent maternal symptoms were headaches (38.5%), cough (30.8%), fever (30.8%), musculoskeletal pain (30.8%), and shortness of breath (23.1%); malaise (15.4%), decreased taste (15.4%), sore throat (7.7%), and diarrhea (8.3%), were the least frequent. Of the 13 newborns from mothers infected with SARS-CoV-2, 61.5% were boys. The average birth weight was 2227 ± 1396 g. The mean Apgar score at 5 min was 7 ± 3. None of the newborns tested positive for SARS-CoV-2 in the first 24 h after birth; one newborn tested positive 7 days after birth (1/13). Despite testing negative in the first 24 h, one of the newborns had shortness of breath after birth. Newborns followed up a week after birth were being breastfed by their mothers.

### 3.3. Characteristics Related to the Transmission of SARS-CoV-2 in Newborns and Maternal or Neonatal Mortality 

Regarding neonatal mortality, two (15.4%) deaths were observed in the newborns. Mortality was related to the delivery category (*p* = 0.021) since the two newborns that died were cesarean deliveries from symptomatic COVID-19 mothers. One of the deliveries was premature, which resulted in a stillbirth, while the other newborn died due to shortness of breath after the mother died due to the worsening of SARS-CoV-2 infection.

The characteristics related to the transmission of SARS-CoV-2 in newborns and maternal or neonatal mortality are provided in Table 3. The transmission rate of SARS-CoV-2 for newborns on the seventh day of life was 9.1% (1/10). We observed a significant relationship between the transmission of SARS-CoV-2 to newborns on the seventh day after birth and the mother’s place of residence (*p* = 0.026), the mother’s educational level (*p* = 0.026), antenatal follow-up (*p* = 0.001), delivery category (*p* = 0.026), and breastfeeding (*p* = 0.001). Two (15.4%) parturients enrolled in the study died due to complications of SARS-CoV-2 infection. However, although no statistical significance was observed (*p* > 0.05), maternal mortality was 50% in parturients who had no antenatal follow-up, while the frequency of maternal mortality decreased to 9% in parturients who had an antenatal follow-up. On the other hand, maternal mortality was strongly related to the delivery category (*p* = 0.021), without any parturient with vaginal delivery having died, while 50% of parturients undergoing cesarean section died. 

## 4. Discussion

Due to altered physiology and immune functions, women constitute an extremely sensitive group that needs to be monitored in the course of many viral infections [6]. The current state of knowledge about SARS-CoV-2 in pregnant women is insufficient, although previous reports showed that special care is needed for the management of pregnancy and the newborn to minimize the adverse effects on mother and child [11].

There are few studies on the natural history of SARS-CoV-2 infection during pregnancy [5]. To the best of our knowledge, this is the first study assessing SARS-CoV-2 transmission in newborns from Luanda, the capital city and the epicenter of the COVID-19 pandemic in Angola. In the current study, 0.4% of pregnant women tested positive for SARS-CoV-2 at the end of pregnancy (Table 1). Previous studies in other countries identified a 10% prevalence of SARS-CoV-2 infection among pregnant women [15]. The rate of SARS-CoV-2 infection increased from 0.3% in parturients under 20 to 0.4% in parturients aged 20 years and over (Table 1). These results are similar to those observed by a study carried out by our research team in the general population of Luanda, where we observed that the rate, as well as the risk of SARS-CoV-2 infection, increased with increasing age [16,17], leading to the belief that these are people who are in the active stage of life and who perform work or other activities that expose them more frequently to the virus. Our results also corroborate those of previous studies in terms of the decrease in infection (0.6% to 0.1%, *p* = 0.012) and risk of infection (OR: 0.18, *p* = 0.025) in non-urbanized to urbanized areas, respectively (Table 1). The low risk of infection in parturients from urbanized regions was expected since compliance with sanitary measures to reduce the spread of the SARS-CoV-2 (e.g., regular handwashing with soap and water, regular use of face masks, use of hand sanitizers, social distance, and, avoidance of clusters) is higher in urbanized regions compared to non-urbanized regions. In addition, the absence of basic minimal sanitary conditions in non-urbanized regions, especially water, could also be associated with this result.

Although none of the COVID-19 parturients previously had concomitant illnesses, pre-eclampsia (7.7%), urinary tract infections (7.7%), and malaria (15.4%) were observed (Table 2). However, the high frequency of malaria observed shows that we need to explore the relationship between vector-borne diseases and SARS-CoV-2 in Luanda many viral infectious agents, such as dengue, zika, chikungunya, and yellow fever, which are transmitted by vectors, are endemic in Angola’s capital city [18,19].

SARS-CoV-2 infection is typically mild during pregnancy [15]. The clinical symptoms reported by nine out of thirteen COVID-19 parturients (Table 2) are similar to those experienced by non-pregnant COVID-19 patients [20,21,22,23]. The frequently observed symptoms such as headaches, cough, fever, musculoskeletal pain, and shortness of breath (Table 2) are similar to those observed in previous studies performed with pregnant women infected with SARS-CoV-2 in China, the USA, Italy, Iran, and Peru [11,15]. Pregnant women with pneumonia have an increased risk of low-birth-weight babies, premature births, restricted fetal growth, and a 5 min Apgar score < 7 compared to healthy pregnant women [5]. Premature birth that resulted in neonatal death was observed in our study. In addition, the average weight (2226.8 ± 1395.5 g) and Apgar score (7.08 ± 2.63 at 5 min) (Table 2) were below the average observed in newborns of healthy mothers [4]. Our results show that the control of infection in asymptomatic pregnant women should be increased because even though we observed 22% of maternal death from symptomatic COVID-19 parturients, we also observed 25% neonatal death from asymptomatic COVID-19 parturients compared to 11% neonatal death in symptomatic COVID-19 parturients (Table 3). These results show that frequent hygiene measures, the use of a face mask, and distance from the newborn should be adopted whenever possible in mothers with asymptomatic or mildly symptomatic SARS-CoV-2 infection when they are breastfeeding or caring for the newborn. The mechanisms for the transmission of SARS-CoV-2 to newborns remain unclear, although previous studies claimed that transmission happens in the case of mothers with high viral load or those critically affected by SARS-CoV-2 [24]. According to our study, the residence area, the mother’s educational level, the fulfillment of antenatal care, and the delivery category could strongly contribute to the transmission of SARS-CoV-2 and the clinical outcome of the neonate exposed to SARS-CoV-2 (Table 3).

The first case of neonatal SARS-CoV-2 infection was reported in China, although it remains controversial whether the case was due to intrauterine vertical transmission [13]. In our study, none of the nasal swab samples taken from newborns tested positive in the first 24 h after birth, which does not exclude the possibility of vertical transmission, since when the viral load is not high enough, the detection rate of existing methods is limited and false negatives may occur [13]. However, one of the newborns from one cesarean delivery performed on symptomatic COVID-19 parturient tested positive on the seventh day after birth (Table 2), even after he was immediately transferred to one of the isolation rooms in the neonatal nursery after delivery. Whether the case occurred due to vertical transmission of SARS-CoV-2 from mother to child in Angola remains to be confirmed. On the other hand, since the study only screened SARS-CoV-2 in parturients, we cannot exclude the possibility that the newborn had contracted a nosocomial infection since some health professionals had COVID-19 during the study period. Wang et al. [13] reported a case of neonatal COVID-19 infection with pharyngeal swabs testing positive by RT-PCR assay 36 h after cesarean delivery; Dong et al. [12] reported a case of a neonate delivered by cesarean in which SARS-CoV-2 IgG and IgM levels were elevated 2 h after birth; Yu et al. [9] reported the case of a positive RT-PCR test in a newborn 36 h after cesarean delivery. A previous study also observed high levels of IgM antibodies against SARS-CoV-2 2 h after the birth of a newborn from a mother with COVID-19, suggesting that the neonate was infected in utero since IgM antibodies generally do not appear until 3 to 7 days after SARS-CoV-2 infection [12]. These results might indicate the need for high attention when carrying out cesarean deliveries in COVID-19 parturients due to the high risk of infection of newborns. Previous studies suggested the performance of cesarean deliveries among COVID-19 pregnant women due to concerns about perinatal transmission because of the risk of ingesting cervicovaginal secretions or the newborn having contact with infected perineal tissue being higher in vaginal delivery [24]. On the contrary, our results showed a high rate of transmission of SARS-CoV-2 in newborns from cesarean (50%) compared to newborns from vaginal (0%) delivery. In addition, 50% of maternal and neonatal deaths were related to cesarean delivery, indicating that performing a cesarean delivery in COVID-19 parturients in Luanda could pose a high risk of SARS-CoV-2 transmission and maternal/neonatal death. Therefore, our results highlight the need to shorten the delivery time of the fetus as well as minimizing contact between the mother’s bodily fluids and the newborn during cesarean delivery. 

The behavior of SARS-CoV-2 and mother-to-child transmission (MTCT) is still unknown due to limited data concerning the MTCT of COVID-19; thus, breastfeeding a newborn from a mother with COVID-19 divides researchers in this field [25]. Separating babies from their mothers is not recommended by WHO; however, measures such as skin-to-skin contact, rooming-in, and exclusive breastfeeding have been strongly encouraged [25]. None of the newborns breastfed by COVID-19-positive mothers tested positive for SARS-CoV-2 at the end of seven days of exclusive and direct breastfeeding (*p* = 0.001) (Table 3), indicating that breastfeeding of newborns from mothers with COVID-19 could be implemented, provided that hygiene measures or the use of a face mask have been complied with during the handling or feeding of the newborn. Breastfeeding in COVID-19 postpartum women was also strongly recommended by Fernández-Carrasco et al. [26], who indicated that if the health of the mother and the newborn allows, direct breastfeeding should be recommended provided that sanitary measures to control the dissemination of SARS-CoV-2 are considered.

Some potential limiting aspects should be considered when interpreting the results of this study. Firstly, all participants enrolled in the study were at the end of pregnancy. Additionally, the sample size is not representative of pregnant women in Luanda. Moreover, the effect of the infection during the first, second, or third trimester of pregnancy has not been explored and needs further investigation. Secondly, the relationship between the delivery categories and the transmission of SARS-CoV-2 in neonates, and maternal and neonatal mortality was not fully explored and needs further studies. Finally, vaginal mucosa and breast milk samples were not collected for the SARS-CoV-2 screening to determine the possibility of SARS-CoV-2 transmission during vaginal delivery or breastfeeding. None of the parturients were vaccinated against SARS-CoV-2, since vaccination was not available at the time of the study; therefore, the effect of vaccination against SARS-CoV-2 infection was not evaluated. Despite the observed limitations, we think that the findings reported here are important for understanding the characteristics of SARS-CoV-2 infection in pregnant women, as well as factors related to transmission to newborns. Our findings also provide useful information for understanding the MTCT of SARS-CoV-2 in Angola, a resource-limited country located in Africa. Therefore, future follow-up studies including pregnant women infected with SARS-CoV-2 and neonates exposed will be needed to help define management strategies for pregnant women and newborns exposed to SARS-CoV-2 in Angola. 

## 5. Conclusions

SARS-CoV-2 infection was relatively low in parturients and newborns regardless of the severity of maternal disease. Our findings suggest that breastfeeding could be safe even for COVID-19-positive mothers. We conducted an early assessment of the outcomes of COVID-19 in late pregnancy, indicating that intensive management of COVID-19 cases in pregnant women could be the best practice in the absence of robust data in Angola.

## Figures and Tables

**Table 1:** Sociodemographic characterization and risk factors of SARS-CoV-2 infection among parturients from Luanda, Angola.

Characteristics	N (%)	SARS-CoV-2 Prevalence			Univariate Analysis		Multivariate Analysis ^#^	
		No (%)	Yes (%)	*p*-Value	OR (95% CI)	*p*-Value	AOR (95% CI)	*p*-Value
Overall	3633 (100)	3620 (99.6)	13 (0.4)					
Age groups								
<20 years	379 (10.4)	378 (99.7)	1 (0.3)	0.799	1.00	-	1.00	-
20–40 years	3166 (87.1)	3154 (99.6)	12 (0.4)		1.44 (0.19–11.1)	0.727	1.48 (0.19–11.8)	0.711
>40 years	88 (2.4)	88 (100)	0 (0.0)		0 (0.0–0.0)	0.997	0 (0.0–0.0)	0.997
Residence area								
Rural	1807 (49.7)	1796 (99.4)	11 (0.6)	**0.012**	1.00	-	1.00	-
Urban	1826 (50.3)	1824 (99.9)	2 (0.1)		0.18 (0.40–0.81)	**0.025**	0.18 (0.04–0.81)	**0.025**
Educational level								
Low	1157 (31.8)	1154 (99.7)	3 (0.3)	0.497	1.00	-	1.00	-
High	2476 (68.2)	2466 (99.6)	10 (0.4)		1.56 (0.43–5.68)	0.500	1.77 (0.47–6.78)	0.402
Occupation								
Unemployed	2599 (71.5)	2588 (99.6)	11 (0.4)	0.295	1.00	-	1.00	-
Employed	1034 (28.5)	1032 (99.8)	2 (0.2)		0.46 (0.10–2.06)	0.307	0.42 (0.09–1.93)	0.264
Antenatal care								
No	419 (11.5)	417 (99.5)	2 (0.5)	0.663	1.00	-	1.00	-
Yes	3214 (88.5)	3203 (99.7)	11 (0.3)		0.72 (0.16–3.24)	0.665	0.65 (0.14–3.07)	0.587

Observation: Bold results mean they were significant in the chi-square or logistic regression (*p* < 0.05). ^#^ Adjusted for all the explanatory variables listed.

**Table 2:** Characteristics of the mother with SARS-CoV-2 infection and neonates born to SARS-CoV-2-infected mothers in Luanda, Angola.

Characteristics	N (%)
Characteristics of the mother with SARS-CoV-2 infection	
No. of mothers	13 (100)
Age (years), mean ± SD	29.9 ± 5.20
Morbidities during pregnancy (other than SARS-CoV-2 infection)	5 (38.5)
Pre-eclampsia	1 (7.7)
Urinary tract infections	1 (7.7)
Malaria	2 (15.4)
Mode of delivery	
Vaginal	9 (69.2)
Cesarean	4 (30.8)
Symptoms related to SARS-CoV-2 infection at the time of delivery	9 (69.2)
Headaches	5 (38.5)
Cough	4 (30.8)
Fever	4 (30.8)
Musculoskeletal pain	4 (30.8)
Shortness of breath	3 (23.1)
Malaise	2 (15.4)
Decreased taste	2 (15.4)
Sore throat	1 (7.7)
Diarrhea	1 (7.7)
Characteristics of the neonates born to SARS-CoV-2-infected mothers	
No. of neonates	13 (100)
Boys	8 (61.5)
Birth weight, mean (SD), g	2226.8 ± 1395.5
Apgar score at 5 min, mean (SD)	7.08 ± 2.63
SARS-CoV-2 positivity	1 (7.7)
At birth	0 (0.0)
At 7 days of life	1 (9.10)
SARS-CoV-2-related symptoms	
At birth	1 (7.7)
At 7 days of life	0 (0.0)
Type of feeding	
Exclusive breastfeeding	10 (90.9)
Exclusive formula milk	1 (9.1)
Breastfeeding and formula milk	0 (0.0)

**Table 3:** Characteristics related to the transmission of SARS-CoV-2 in newborns and maternal or neonatal mortality related to COVID-19 in Luanda, Angola.

Characteristics of the Mothers	Transmission of SARS-CoV-2 to Newborns at Day 7			Maternal Death			Neonatal Death		
	No (%)	Yes (%)	*p*-Value	No (%)	Yes (%)	*p*-Value	No (%)	Yes (%)	*p*-Value
Overall	10 (90.9)	1 (9.10)		11 (84.6)	2 (15.4)		11 (84.6)	2 (15.4)	
Age groups									
<20 years	1 (100)	0 (0.0)	0.740	1 (100)	0 (0.0)	0.657	1 (100)	0 (0.0)	0.657
≥20 years	9 (90.0)	1 (10.0)		10 (83.3)	2 (16.7)		10 (83.3)	2 (16.7)	
Residence area									
Rural	9 (100)	0 (0.0)	**0.026**	10 (90.9)	1 (9.1)	0.140	9 (81.8)	2 (18.2)	0.512
Urban	1 (50.0)	1 (50.0)		1 (50.0)	1 (50.0)		2 (100)	0 (0.0)	
Educational level									
Low	1 (50.0)	1 (50.0)	**0.026**	2 (66.7)	1 (33.3)	0.326	2 (66.7)	1 (33.3)	0.326
High	9 (100)	0 (0.0)		9 (90.0)	1 (10.0)		9 (90.0)	1 (10.0)	
Occupation									
Unemployed	8 (88.9)	1 (11.1)	0.621	9 (81.8)	2 (18.2)	0.512	9 (81.8)	2 (18.2)	0.512
Employed	2 (100)	0 (0.0)		2 (100)	0 (0.0)		2 (100)	0 (0.0)	
Antenatal care									
No	0 (0.0)	1 (100)	**0.001**	1 (50.0)	1 (50.0)	0.140	1 (50.0)	1 (50.0)	0.140
Yes	10 (100)	0 (0.0)		10 (90.9)	1 (9.1)		10 (90.9)	1 (9.1)	
Symptomatic disease									
No	3 (100)	0 (0.0)	0.521	4 (100)	0 (0.0)	0.305	3 (75.0)	1 (25.0)	0.522
Yes	7 (87.5)	1 (12.5)		7 (77.8)	2 (22.2)		8 (88.9)	1 (11.1)	
Delivery category									
Vaginal	9 (100)	0 (0.0)	**0.026**	9 (100)	0 (0.0)	**0.021**	9 (100)	0 (0.0)	**0.021**
Cesarean	1 (50.0)	1 (50.0)		2 (50.0)	2 (50.0)		2 (50.0)	2 (50.0)	
Exclusive breastfeeding									
No	0 (0.0)	1 (100)	**0.001**	0 (0.0)	1 (100)	**0.001**	1 (100)	0 (0.0)	-
Yes	10 (100)	0 (0.0)		10 (100)	0 (0.0)		10 (100)	0 (0.0)	

Observation: Bold results mean they were significant in the chi-square test.

## Data Availability

The datasets used and/or analyzed during the current study are available from the corresponding author on reasonable request.
